# Canadian guidelines for rhinosinusitis: practical tools for the busy clinician

**DOI:** 10.1186/1472-6815-12-1

**Published:** 2012-02-01

**Authors:** Shaun Kilty

**Affiliations:** 1 Department of Otolaryngology-Head and Neck Surgery, The Ottawa Hospital, University of Ottawa, ON, Canada

## Abstract

Acute bacterial rhinosinusitis (ABRS) and chronic rhinosinusitis (CRS) frequently present in clinical practice. Guidelines for management of these conditions have been published extensively in the past. However, a set of guidelines that addressed issues specific to the Canadian environment while offering clear guidance for first-line clinicians was needed, and resulted in the recent publication of Canadian clinical practice guidelines for ABRS and CRS. In addition to addressing issues specific to Canadian physicians, the presented guidelines are applicable internationally, and offer single algorithms for the diagnosis and management of ABRS and CRS, as well as expert opinion in areas that do not have an extensive evidence base. This commentary presents major points from the guidelines, as well as the intended impact of the guidelines on clinical practice.

See guidelines at: http://www.aacijournal.com/content/7/1/2

## Introduction

The first comprehensive Canadian clinical practice guidelines for acute bacterial rhinosinusitis (ABRS) and chronic rhinosinusitis (CRS) have recently been co-published in *Allergy Asthma Clin Immunol *[1] and in *J Otolaryngol Head Neck Surg *[2]. Although rhinosinusitis guidelines have been issued by European and American medical societies over the last few years [3-6], and guidance for the Canadian environment has appeared [7,8], the current Canadian guidelines mark the first time that comprehensive guidelines covering both ABRS and CRS appear, with a focus on addressing issues specific to the Canadian healthcare environment.

The term rhinosinusitis is used to denote inflammation of the sinus and nasal passages, which often occur simultaneously due to their close location and shared respiratory epithelium. Rhinosinusitis is common and increasing in prevalence worldwide. It is associated with a significant burden on healthcare services, quality of patients' lives, and lost patient productivity. The prevalence of acute rhinosinusitis increased from 11% (or 26 million) of American adults in 2006 [9] to nearly 13% (over 29 million) in 2009 [10]. Nearly 7 years ago, the economic burden of the cost of treatment was estimated at 6 billion dollars annually in the United States [11]. Clearly, as rhinosinusitis continues to affect more individuals, the impact on patient lives and total costs will also continue to rise.

## Discussion

**Figure 1:**
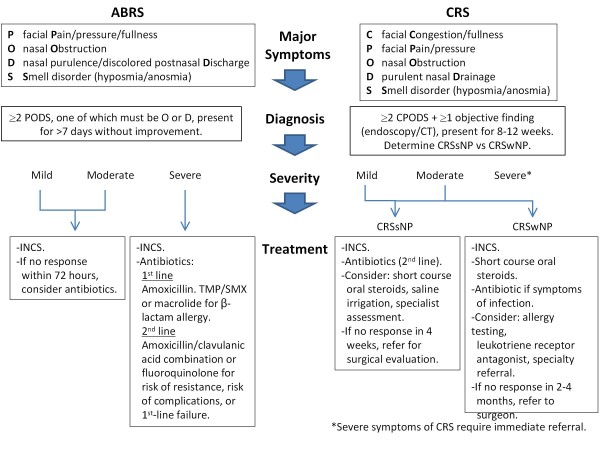
**Overview of algorithms for ABRS and CRS**.

Because ABRS and CRS have different pathologies and thus management strategies, it is critical that clinicians understand these differences so appropriate treatment can be started. However, analysis of Canadian prescription data demonstrated nearly identical prescribing habits for patients with ABRS and for those with CRS [12], highlighting that differences in the treatment of these distinct conditions were not fully appreciated. The Canadian guidelines provide easy-to-read and practical recommendations to assist clinicians who face patients with symptoms of rhinosinusitis in everyday practice. These guidelines provide specific updates on a variety of topics, including diagnostic symptom duration and severity, choice of treatment, appropriate testing, and antimicrobial resistance issues, in addition to providing useful diagnostic tools. An overview of the diagnostic and treatment algorithms is presented in Figure 1.

### ABRS diagnosis

The diagnosis of ABRS requires the duration of appropriate symptoms be greater than 7 days. Prior guidelines suggest 5 days with worsening symptoms or 10 days of persistent symptoms as the lower end of duration [3], or symptoms that persist more than 10 days after viral symptoms present, or worsen within 10 days of initial improvement [5]. Although symptoms of viral infections may linger, they reach peak severity by 3 days [13,14]. Thus, the current guidelines recommend that ABRS be considered for symptoms lasting longer than 7 days without improvement, or for symptoms that worsens after 5 to 7 days, or for symptoms that persist after 10 days.

A unique offering of these guidelines is the mnemonic, PODS, to assist clinicians in recalling the major symptoms of ABRS. PODS stands for facial Pain/pressure/fullness, nasal Obstruction, nasal purulence/postnasal Discharge, and Smell alterations (hyposmia/anosmia). For a diagnosis, at least 2 of these major symptoms must be present, one of which must be O (obstruction) or D (discharge). To my knowledge, this is the first set of guidelines offering such a mnemonic for the major diagnostic criteria of ABRS, which should help clinicians quickly assess the information that is needed for a diagnosis. The guidelines also provide a discussion of prediction rules that describe symptoms whose cumulative presence increases the likelihood of bacterial infection. In the absence of diagnostic tests, ABRS diagnosis relies on clinical symptoms coupled with duration despite challenges in the accuracy and specificity of signs and symptoms.

### ABRS treatment

Symptom severity plays a role in determining appropriate therapeutic intervention. Previous guidelines have defined symptom severity either on scores from a visual analogue score [3] or based upon temperature and pain severity [5]. The Canadian guidelines base severity by the degree to which symptoms impair the patient. Thus, low severity is defined as easily tolerated symptoms, moderate severity reflects steady symptoms that are tolerable, and severe severity indicates that symptoms are difficult to tolerate or interfere with sleep or daily activities. This approach does not depend on the presence of fever, which is not included as a major symptom of ABRS. Symptom severity is then used to determine therapeutic intervention. For symptoms that are mild to moderate in intensity, the guidelines recommend the use of intranasal corticosteroids (INCS)--but not antibiotics--as a first step in treatment. In mild to moderate severity illness, antibiotics are reserved for patients who fail to respond to INCS after 3 days and whose symptoms continue for more than 7 days. For patients presenting with severe illness, INCS and antibiotics are recommended in combination as a first step in treatment. This approach thus reserves antibiotics for clear cases of ABRS that are not resolving spontaneously.

In general, amoxicillin remains the first-line choice for ABRS, with trimethoprim/sulfamethoxazole (TMP/SMX) or macrolides recommended for individuals with β-lactam allergy. However, antibiotic choice depends upon other considerations as well, including local antimicrobial resistance patterns, patient risk of resistance, and risk of complications of failure due to underlying disease. For patients with risk of resistance or complications of first-line failure, a second-line agent (amoxicillin/clavulanic acid combinations, fluoroquinolones) is recommended.

### CRS diagnosis

A useful mnemonic for quick recall of major symptoms of CRS is CPODS, standing for facial Congestion/fullness, facial Pain/pressure, nasal Obstruction/blockage, nasal Drainage, and Smell dysfunction (hyposmia/anosmia). At least two of the CPODS symptoms must be present for 8 to 12 weeks, along with documented inflammation of the nasal mucosa or paranasal sinuses. The duration of symptoms for diagnosis has generally been proposed as 12 weeks [3,5]. However, Canadian guidelines align with others in recommending an 8 week minimum for CRS diagnosis [15]. The guidelines also highlight the insistence of clinical or radiologic evidence in addition to clinical symptoms in order to make a diagnosis of CRS. Hence, a diagnosis requires the presence of 2 or more CPODS, present for 8 to 12 weeks, plus objective evidence using either endoscopy or computed tomography. A recent study of EP3OS symptom criteria confirmed by endoscopy reported moderate reliability of symptom-based diagnosis for CRS, and found stability between geographic regions [16]. These findings concur with an earlier study that found that endoscopy was critical to increase the specificity, and positive and negative predictive values for symptom-based diagnosis of CRS [17]. Although symptom-based diagnosis had poor specificity by itself, diagnostic accuracy improved dramatically when combined with an objective finding (odds ratio for CRS improved from 1.1 to 4.6 [95% CI, 2.3-9.2]).

The presence or absence of nasal polyps is used to further categorize disease. The presence of bilateral polyps in the middle meatus characterizes CRS with nasal polyps (CRSwNP), while the lack of polyps constitutes CRS without nasal polyps (CRSsNP). The guidelines provide detailed description of the physical examination, including adequate tools for visual rhinoscopy assessments and description of clinical signs, which should provide clinicians with a better understanding of useful signs and symptoms.

### CRS treatment

Because CRS is primarily an inflammatory disease with unknown contributions from bacteria, cytokines, leukocytes, and tissue remodeling, treatment is based upon use of INCS as monotherapy or as adjunct therapy with antibiotics. Before initiating one of these treatments, predisposing and contributing conditions should be identified and treated. Conditions that are thought to contribute to CRS include allergic rhinitis, asthma, ciliary dysfunction, immune dysfunction, lost ostia patency, aspirin-exacerbated respiratory disease, and cystic fibrosis.

Nasal or oral corticosteroids are used with or without antibiotics for initial treatment of CRSsNP. When prescribed, antibiotics should be a second-line agent with broad-spectrum coverage (ie, amoxicillin-clavulanic acid inhibitors, fluoroquinolones) and duration of therapy should be longer than for ABRS.

For CRSwNP, a course of topical INCS and short courses of oral steroids are used. Antibiotics are not recommended for CRSwNP unless there are symptoms suggesting infection.

### Role of antibiotics

These guidelines discuss concerns of increasing rates of antibiotic resistance, with the emergence of penicillin, macrolide, and multi-drug resistant *S. pneumoniae*. Elevated rates of β-lactamase-producing *H. influenzae *and *M. catarrhalis *have also been reported, with consequences for antibiotic choice. Although antibiotic resistance rates increased between 1988 and 2005, some rates have stabilized in the 5-year period between 2000 and 2005, including rates for ciprofloxacin, penicillin, and TMP/SMX resistance [18,19]. However, resistance rates continue to increase for erythromycin and tetracycline, and penicillin nonsusceptibility remains elevated. Resistance and nonsusceptibility rates also showed regional differences. Similar patterns of variable and growing antibiotic resistance are of global concern, with the World Health Organization dedicating World Health Day 2011 to the topic of antimicrobial resistance. Because of the trends in resistance rates, guidelines advise judicious use of antibiotic therapy and awareness of related issues, with appropriate selection of therapy for clinical situation.

### Ancillary therapy, testing, and prevention

Saline irrigation has emerged in these guidelines as a strong recommendation from the experts despite limited evidence. This expert endorsement is in line with the increased recognition of the benefits of saline therapy over the last 5 to10 years in the management of sinonasal disease. Multiple studies report the benefit of saline irrigation in patients with CRS [20-22], ARS [23], and in allergic, acute and chronic rhinosinusitis [24]. The use of saline irrigation as adjunct therapy is primarily based upon reported symptomatic improvement and its good safety profile.

The expert panel endorsed allergy testing in cases of recurrent episodes of ABRS or for CRS as a potential contributing condition to these diseases. Indeed, studies have reported that over half of patients with recurrent ARS or with CRS tested positive for allergies [25,26]. Whether allergy treatment helps minimize symptoms in these cases is not known, but knowledge and avoidance of identified allergens remains a prudent approach none the less.

Prevention of illness is a recommendation. The importance of hand washing is highlighted as fundamental to reduce viral transmission and prevent the development of ABRS and acute exacerbations of CRS. Although studies are lacking, the expert recommendation strongly encourages educating patients regarding strategies to reduce the likelihood of contracting a viral illness.

### Guideline rationale and method

The Canadian clinical practice guidelines for acute and chronic rhinosinusitis were designed foremost to be a valuable tool for first-line clinicians, particularly primary care providers. As such, the guidelines are evidence based, make specific recommendations, and comprehensively address both ABRS and CRS in single algorithms for each, while also providing the practitioner with the opinion of clinical experts.

Previous Canadian guidelines were either outdated or did not provide guidance for practitioners in best practices management of patients with ABRS or CRS. The current guidelines present strategies relevant for Canadian practitioners to negotiate the unique aspects of their healthcare system, while remaining sufficiently general to benefit an international audience. Thus, in situations that might arise in the Canadian healthcare system, such as delays in obtaining a computed tomography scan or specialist referral, action points during this waiting period are presented. However, the statements and discussions throughout the document focus on aspects central to ABRS and CRS, and apply in any healthcare environment. Advances in the pathobiology of these disease entities are discussed, along with the current status of treatment options.

The guidelines were constructed using an evidence-based strategy and present an evidence strength rating, plus a second rating that reflects the endorsement level of the multidisciplinary group of experts who represent medical fields involved in treating this multifaceted condition. This latter rating is particularly useful in areas where rigorous trials are lacking in quality or number. The guidelines were further reviewed and endorsed by the Association of Medical Microbiology and Infectious Disease Canada, Canadian Society of Allergy and Clinical Immunology, Canadian Society of Otolaryngology-Head and Neck Surgery (CSO-HNS), Canadian Association of Emergency Physicians, and the Family Physicians Airways Group of Canada.

The Canadian guidelines provide clear definitions and discussion of ABRS and CRS. The diagnostic and treatment flow diagrams are complete, providing detail and ease of flow within a single algorithm for ABRS and for CRS. The guidelines were designed to improve the 'usability' for general practitioners and family medicine physicians. In addition, these guidelines provide current, clear information for the optimal management of CRS-a disease entity for which advances in understanding its pathobiology and effective management strategies continue to evolve. The steps involved in the diagnosis, management, and referral of ABRS and CRS, coupled with strength of recommendation ratings, should help arm general practitioners with the information necessary to improve the management of their patients with symptoms of rhinosinusitis.

## Conclusion

The knowledge base of ABRS and CRS pathology and management continues to grow and evolve as the role of pathogens are discerned, antimicrobial resistance rates change, and treatment strategies are improved. The Canadian guidelines recognize this changing environment and provide a solid foundation for future developments. The guidelines are a much-needed user-friendly tool for busy physicians who want to quickly grasp the appropriate methods of diagnosis and management of ABRS and CRS. The inclusion of mnemonic devices to aid in diagnosis, succinct algorithms for each disease entity, and expert opinion to aid where studies are lacking create a new level of utility for rhinosinusitis guidelines. Physicians who desire to understand the current state of the pathologies and treatment approaches for ABRS and CRS will benefit from these guidelines, and as an extension, their patients will benefit as well.

## List of abbreviations

ABRS: acute bacterial rhinosinusitis; CRS: chronic rhinosinusitis; INCS: intranasal corticosteroids; TMP/SMX: trimethoprim/sulfamethoxazole.

## Competing interests

The author declares that they have no competing interests.

## Pre-publication history

The pre-publication history for this paper can be accessed here:

http://www.biomedcentral.com/1472-6815/12/1/prepub
